# Whole exome sequencing detects *CHST3* mutation in patient with acute promyelocytic leukemia: A case report

**DOI:** 10.1097/MD.0000000000012214

**Published:** 2018-09-07

**Authors:** Lili Feng, Ying Li, Ying Li, Yujie Jiang, Na Wang, Dai Yuan, Juan Fan

**Affiliations:** a Department of Hematology, Provincial Hospital Affiliated to Shandong University; b Department of Diagnostics, Shandong University School of Medicine, Jinan, China.

**Keywords:** acute promyelocytic leukemia, *CHST3*, skeleton dysplasia, whole exome sequencing

## Abstract

**Rationale:**

Acute promyelocytic leukemia (APL) is a kind of acute myeloid leukemia, which was characterized by the presence of *PML/RARα* fusion gene. Mutations in *CHST3* have been previously reported to be associated with a rare phenotype of skeleton dysplasia, known as Spondyloepiphyseal dysplasia. Here we reported 1 patient with APL with *CHST3* mutations.

**Patient concerns:**

An 18-year-old girl was referred to the Hematology Department because of a lasting history (10 days) of repeated fever and bleeding on skin. The girl was of short stature for age and with short fingers. Double nail beds were short with anti-nail deformity.

**Diagnoses:**

She was diagnosed with APL according to the 2016 WHO classification after a MICM analysis (bone marrow morphology [M], immunophenotype [I], cytogenetics [C], and molecular biology [M]). Whole exome sequencing revealed complex heterozygous mutations on *CHST3*. Further confirmation showed that 1 mutation (c.155T>G; p.Leu52Arg) was from her father and the other mutation (c.1414G>A; p.Glu472Lys) was from her mother.

**Interventions:**

The patient received Idarubicin (8 mg/m^2^) injection intravenous drip for 3 days based on all-trans retinoic acid and arsenic trioxide induction therapy.

**Outcomes:**

The patient died from disseminated intravascular coagulation and multiple organ hemorrhage at 9 days after diagnosis.

**Lessons:**

This case describes a patient with APL with complex heterozygous mutations on *CHST3*. Carbohydrate sulfotransferases were found to play an important role in metastatic spread of tumor cells. Whether the mutation status of *CHST3* gene has relationship with APL pathogenesis and prognosis is unknown.

## 1 Introduction

Acute promyelocytic leukemia (APL) is a special kind of acute myeloid leukemia (AML) characterized by the presence of *PML/RARα* fusion gene located on chromosome 15 and chromosome 17, respectively. With the introduction of all-trans retinoic acid (ATRA) and arsenic trioxide (ATO)-containing regimens in APL therapy, APL is now characterized by complete remission rates of 90% and cure rates of ∼80%, even higher among low-risk patients.^[1]^ Nevertheless, APL remains associated with a significant incidence of early death related to the characteristic bleeding diathesis. Early death has emerged as the major cause of treatment failure. Mutations in *CHST3* have been previously reported to be associated with a rare phenotype of skeleton dysplasia, known as Spondyloepiphyseal dysplasia with congenital joint dislocations which is an autosomal recessive inherited disease.^[2]^ Here we reported 1 patient with APL with complex heterozygous mutations on *CHST3*. Further confirmation showed that mutations were from her parents, respectively. Her parents were not close relatives and family members had no similar deformity. Whether the mutation status of *CHST3* gene will increase the formation the *PML/RARα* fusion gene or relate with early death of APL is unclear now.

## 2 Case presentation

This case report conformed to the principles of the Declaration of Helsinki. Study procedures were approved by the Ethics Committee of the Provincial Hospital Affiliated to Shandong University, China. Informed consent was obtained from the patient and her parents for publication of this case report and accompanying images. An 18-year-old girl was referred to the Hematology Department because of a lasting history (10 days) of repeated fever and bleeding on skin, without evidence of inflammatory, systemic, allergic, or autoimmune diseases. A complete blood count values were the following: hemoglobin, 86 g/L (normal reference value 115.0–150.0 g/L); leukocyte count, 31.01 × 10^9^/L (normal reference value 4.0–10.0 × 10^9^/L); and platelet count, 34 × 10^9^/L (normal reference value 100–300 × 10^9^/L). Further morphologic examination of peripheral blood smear identified that infantile cells account for 30% of total leukocytes. Bone marrow aspirate and biopsy was taken from the right ileac crest of the patient. Bone marrow smears demonstrated that 93% abnormal cells were leukoerythroblastosis. Immunophenotyping by flow cytometry showed that leukemia cells were positive for CD13, CD33, CD64, CD38, CD9, cMPO, partial cells expressed CD117, whereas negative for CD5, CD19, CD34, HLA-DR, CD7, CD10, CD15, CD11b, CD14, CD56, CD20, GlyA, cCD79a, and cCD3. Chromosome analysis of bone marrow indicated a complex karyotype of APL (46,XX,t (15;17)(q24;q21)[13]/46,XX,der (15)t (15;17)(q24;q21), ider (17)(q10)t (15;17)(q24;q21)[7]). Polymerase chain reaction analysis with BCR/ABL, PML/RARA, AML1/ETO, CBFβ/MYH11, MLL/AF9, MLL/AF10, MLL/ELL, MLL/AF17, MLL/AF6, NPM/RARA, PLZF/RARA, AML1-MDS1/EVI1, NPM/MLF1, STAT5b/RARA, NUP98/HOXA9, NUP98/HOXA11 primes showed *PML/RARα* fusion gene was positive. Based on these results, the patient was diagnosed with APL. The patient received Idarubicin (8 mg/m^2^) injection intravenous drip for 3 days based on ATRA (25 mg/m^2^/d) and ATO (0.16 mg/kg/d) induction therapy. Unfortunately, she died from disseminated intravascular coagulation and multiple organ hemorrhage at 9 days after diagnosis though positive therapies were carried out.

**Figure 1:**
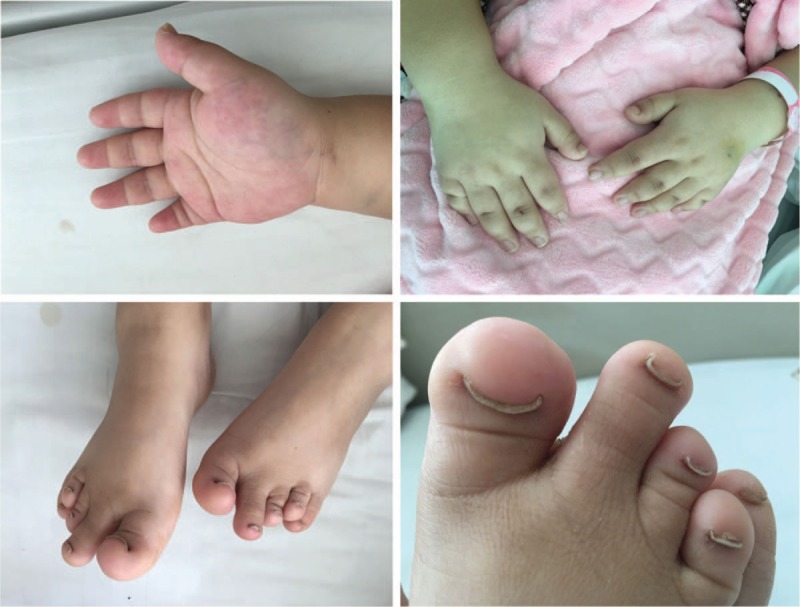
The patient has short fingers and double nail beds were short with anti-nail deformity.

**Figure 2:**
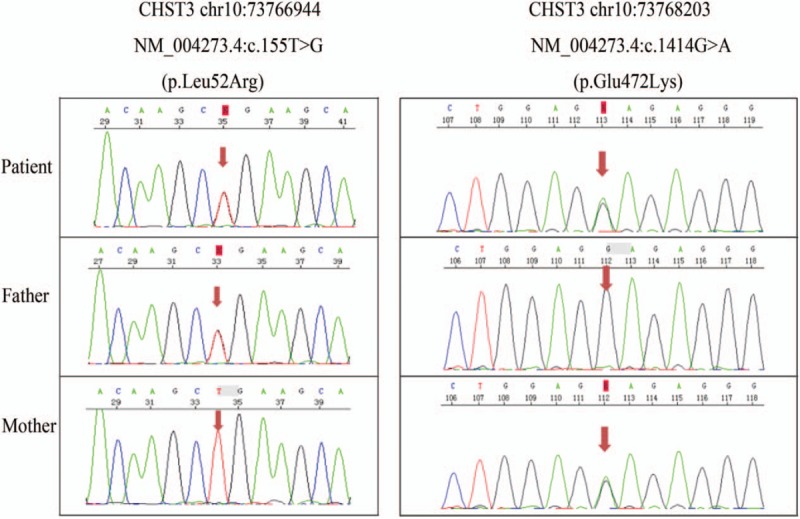
Sanger sequencing confirmed that one mutation (c.155T>G; p.Leu52Arg) was from her father and the other mutation (c.1414G>A; p.Glu472Lys) was from her mother.

Meanwhile, the girl was of short stature for age (height: 140 cm) and with short fingers. Double nail beds were short with anti-nail deformity (Fig. 1). Her parents are not close relatives. The girl has 1 younger sister. Her father has 1 younger brother and 3 younger sisters. Her mother has 4 elder brothers. The brothers and sisters of her parents have 20 children totally. However, her family members have no similar deformity. Whole exome sequencing (WES) was then performed using genomic DNA samples from the patient's peripheral blood. Genomic DNAs were also extracted from her parents’ peripheral bloods for further confirmation. We found that the patient had complex heterozygous mutations on *CHST3* gene. Further confirmation showed that one mutation (c.155T>G; p.Leu52Arg) was from her father and the other mutation (c.1414G>A; p.Glu472Lys) was from her mother. Sanger sequencing was also confirmed the results (Fig. 2).

## 3 Discussion

The APL is 1 kind of AML characterized by the balanced reciprocal translocation between the promyelocytic leukemia gene on chromosome 15 and the retinoic acid receptor α (*RARα*) gene on chromosome 17, and accounts for 10% to 15% of newly diagnosed AML each year. The combined use of ATRA and ATO as primary therapy has markedly improved the survival rate of patients with APL.^[3]^ However, mortality in the first 30 days following therapy remains a major contribution to treatment failure. It was reported that immunophenotyping and complex karyotype maybe help us to identify high-risk patients of early death.^[3]^ CD56^+^ and CD34^+^/CD2^+^ may be candidates to select high-risk patients. While in this patient, leukemia cells were negative for CD56 or CD34. The girl owned a complex karyotype which maybe partially responsible for her early death. In another study, Xu et al analyzed 212 newly diagnosed patients with APL and they found that a high WBC count contributes to the occurrence of more early death, which is usually not associated with delay of diagnosis and hospitalization.^[4]^ For our patient, the WBC number at diagnosis was more than 10 × 10^9^/L, which may also contributed to the early death of this patient. However, were there any other factors to participate in her early death were still unknown.

Sulfation is a critical modification in many instances of biologic recognition. Carbohydrate sulfotransferases (CHSTs) are the enzymes that transfer sulfate to carbohydrate groups in glycoproteins. CHST family is responsible for sulfation of dermatan, keratan, and chondroitin sulfate structures. Mutations in *CHST3* gene have been associated with skeleton dysplasia, which is an autosomal recessive inherited disease, primarily among patients with short stature, joint dislocation, and kyphoscoliosis.^[2]^ Other associated phenotypes include microdontia and cardiac valve anomalies.^[2,5]^ Our patient was found to be with short stature, while without joint dislocation, kyphoscoliosis, or microdontia. No cardiac valve abnormalities were detected by B-ultrasound examination. However, the patient had short fingers. Nail beds were also short with anti-nail deformity. WES and Sanger sequencing confirmed that the patient had complex heterozygous mutations on *CHST3* (c.155T>G; p.Leu52Arg and c.1414G>A; p.Glu472Lys) which were from her parents, respectively. Maybe this was the reason that the patient did not have classical performance of skeleton dysplasia.

The CHSTs were also reported to play an important role in metastatic spread of tumor cells. In a study conducted by Oliveira-Ferrer and colleagues, they compared mRNA expression levels of *CHST3/7/11/12/13/15* between malignant and nonmalignant tumors.^[6]^ They found that mRNA expression of *CHST11*, *CHST12*, and *CHST15* was significantly higher in ovarian cancer samples compared with nonmalignant ones. However, for *CHST3* and *CHST7*, no significant differences were found between the 2 groups. And also, high CHST11 protein expression was independently associated with unfavorable progression-free survival. The roles of CHST family in pathogenesis and prognosis of leukemia have not been reported previously. Here we reported 1 APL patient with *CHST3* mutation for the first time. Her mutation (c.155T>G; p.Leu52Arg) and the other mutation (c.1414G>A; p.Glu472Lys) of *CHST3* have neither been reported previously. Whether the mutation status of *CHST3* gene has relationship with the occurrence the *PML/RARα* fusion gene and the early death of APL still needs further exploration.

## Author contributions

**Conceptualization:** Lili Feng.

**Investigation:** Ying Li, Ying Li.

**Methodology:** Yujie Jiang, Na Wang.

**Resources:** Dai Yuan.

**Validation:** Juan Fan.

**Writing – original draft:** Lili Feng.

**Writing – review & editing:** Juan Fan.
